# Supplementary material for: Combined Widely Targeted Metabolomic, Transcriptomic, and Spatial Metabolomic Analysis Reveals the Potential Mechanism of Coloration and Fruit Quality Formation in Actinidia chinensis cv. Hongyang
Source: Foods. 2024 Jan 11;13(2):233. doi: 10.3390/foods13020233 (PMC10814455; doi:10.3390/foods13020233)
Supplement: Supplementary file 1 [file foods-13-00233-s001.zip › Figure S1.pdf]

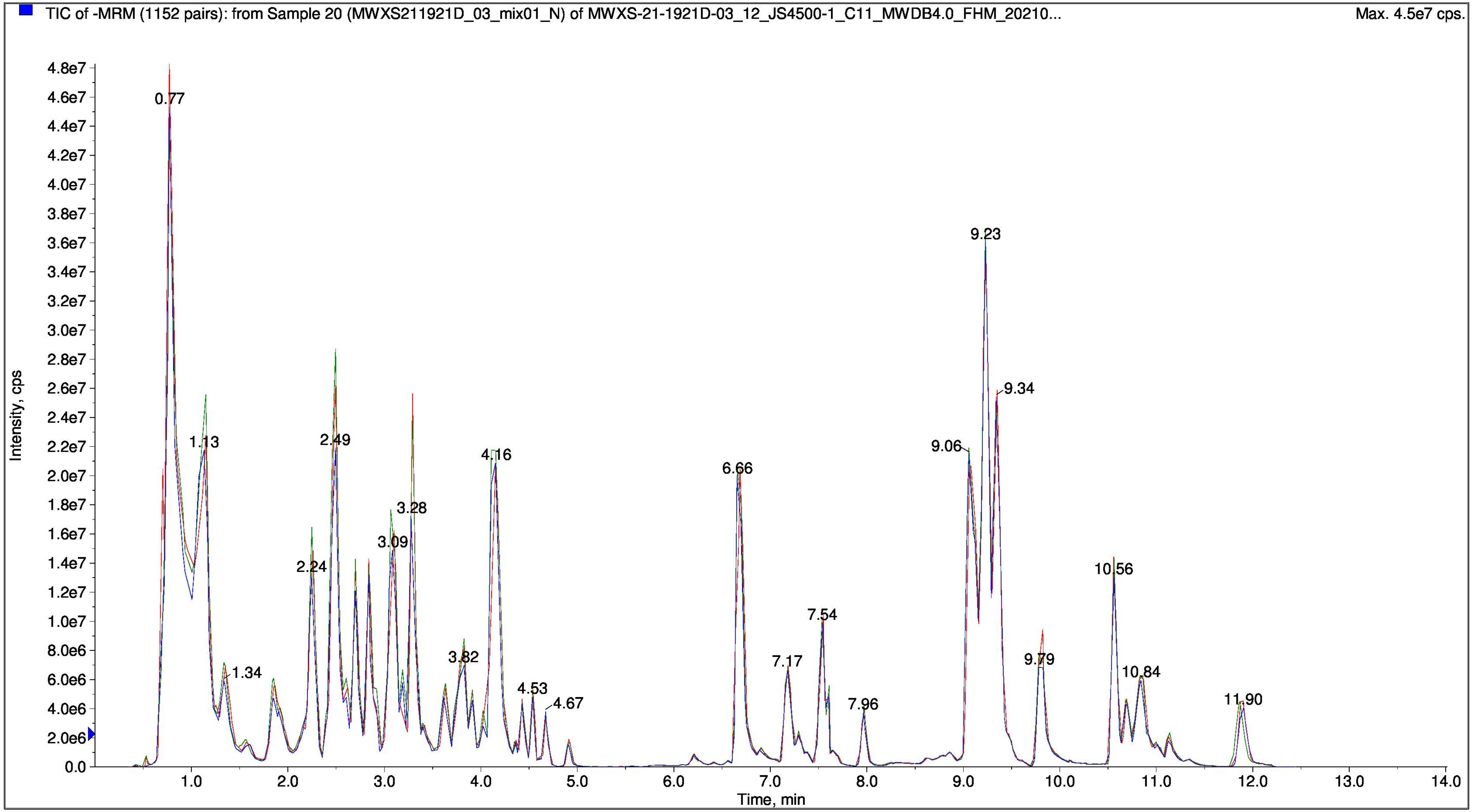

Figure S1-1. Total Ion Current of quality control samples in negative ion mode

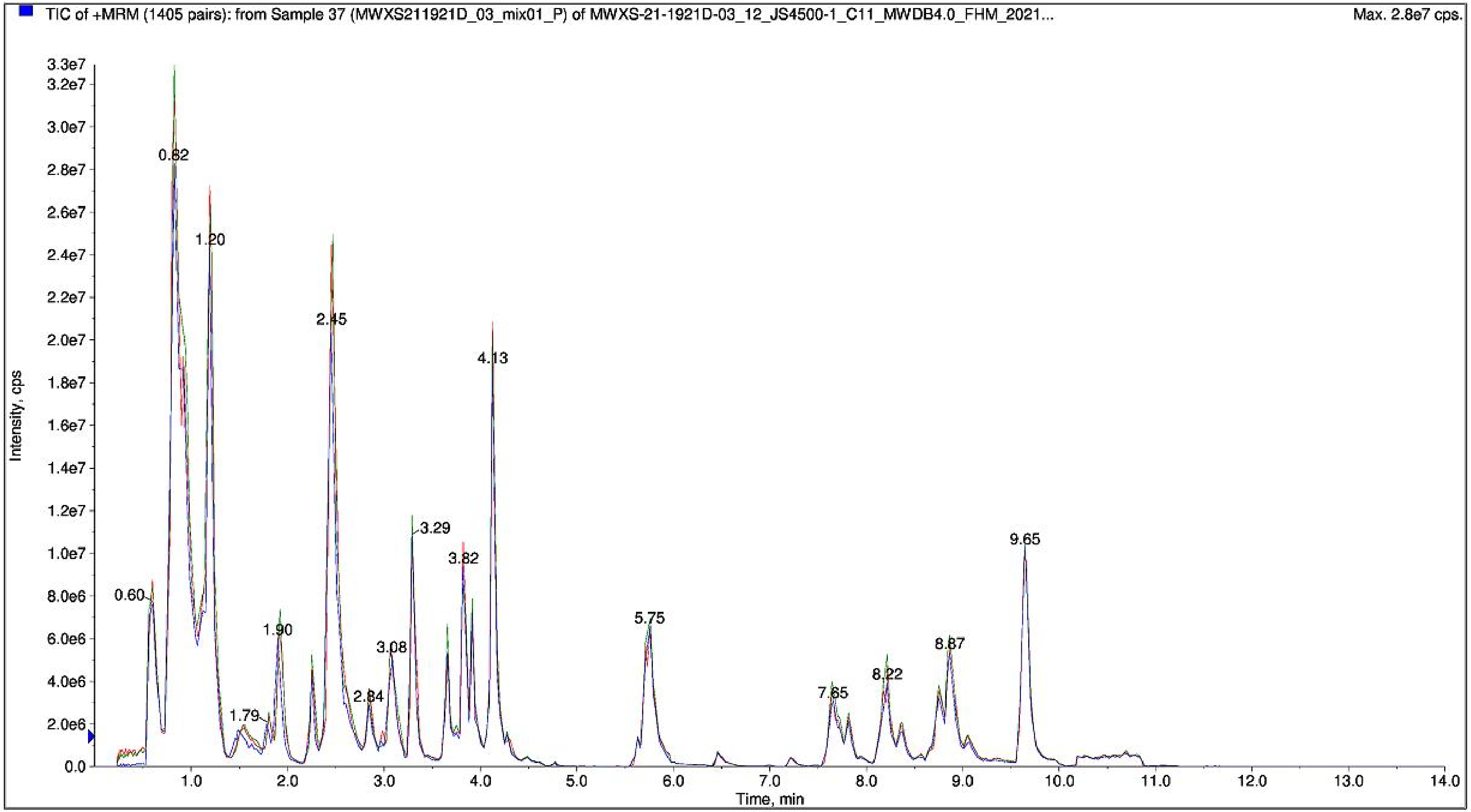

Figure S1-2. Total Ion Current of quality control samples in positive ion mode
