# Supplementary material for: Combined Widely Targeted Metabolomic, Transcriptomic, and Spatial Metabolomic Analysis Reveals the Potential Mechanism of Coloration and Fruit Quality Formation in Actinidia chinensis cv. Hongyang
Source: Foods. 2024 Jan 11;13(2):233. doi: 10.3390/foods13020233 (PMC10814455; doi:10.3390/foods13020233)
Supplement: Supplementary file 1 [file foods-13-00233-s001.zip › Figure S2.pdf]

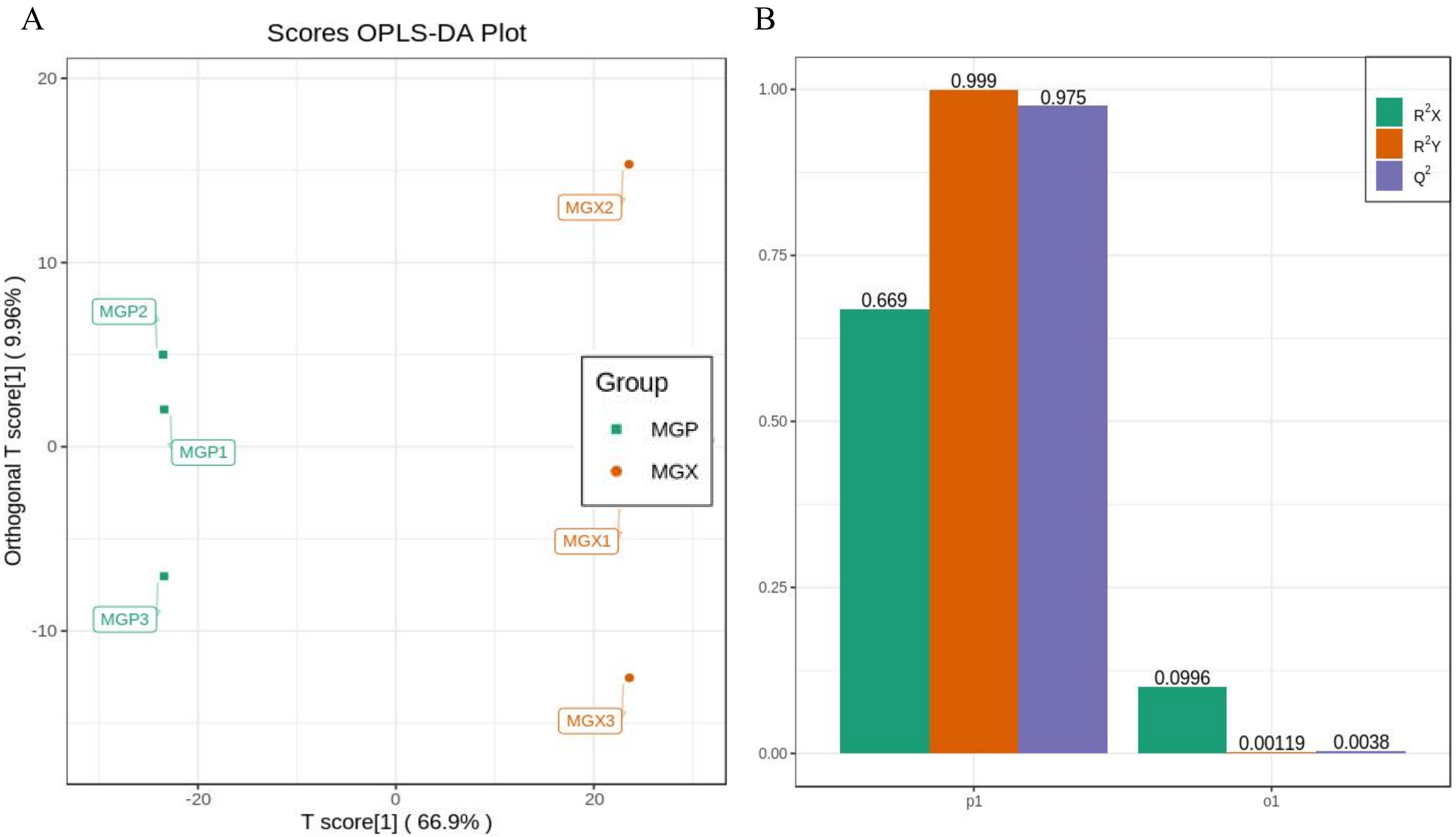

Figure S2-1. Orthogonal partial least squares-discriminant analysis (OPLS-DA) (A) and 200-response sorting tests of MGP_vs_MGX (B).

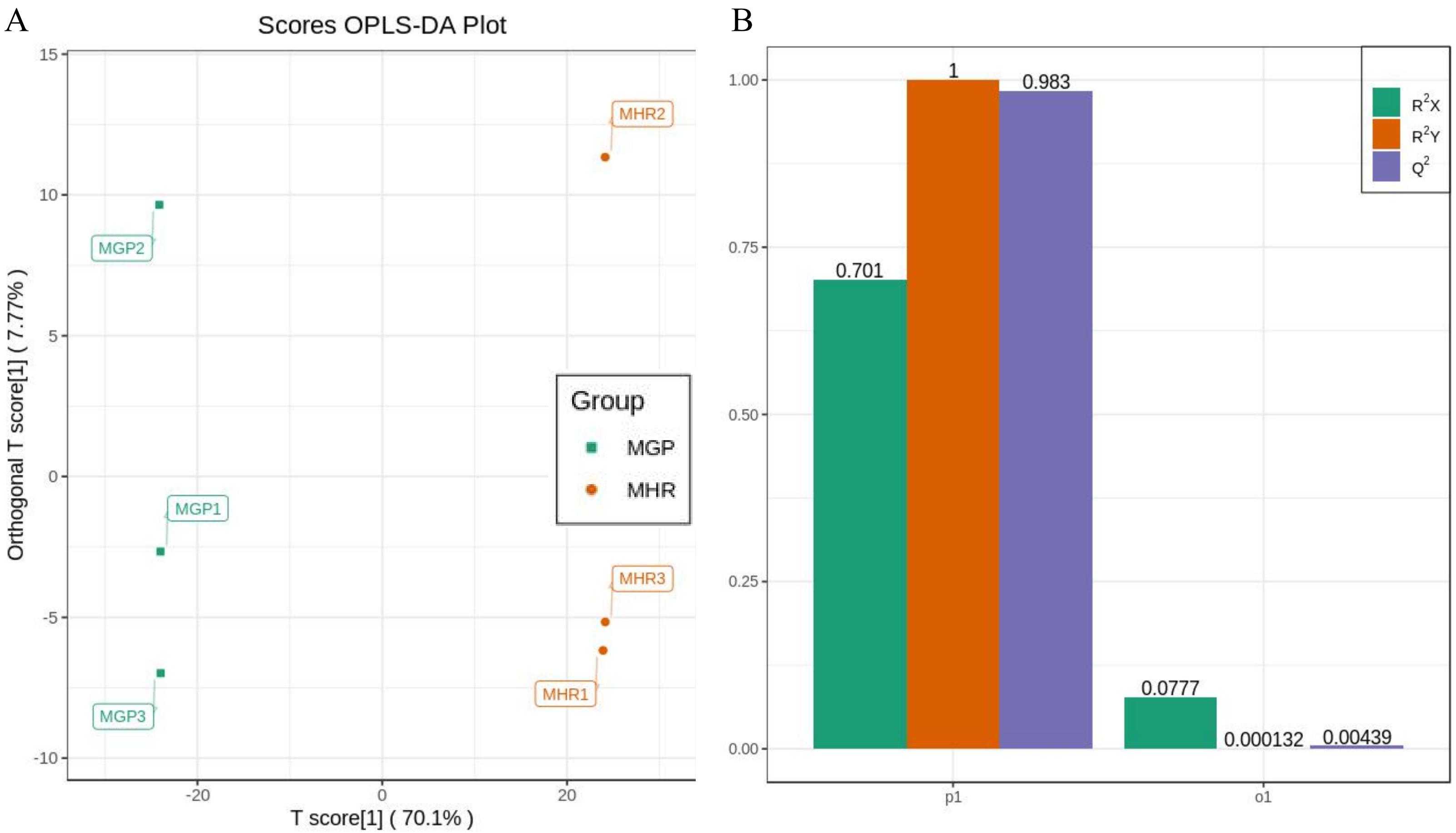

Figure S2-2. Orthogonal partial least squares-discriminant analysis (OPLS-DA) (A) and 200-response sorting tests of MGP_vs_MHR (B).

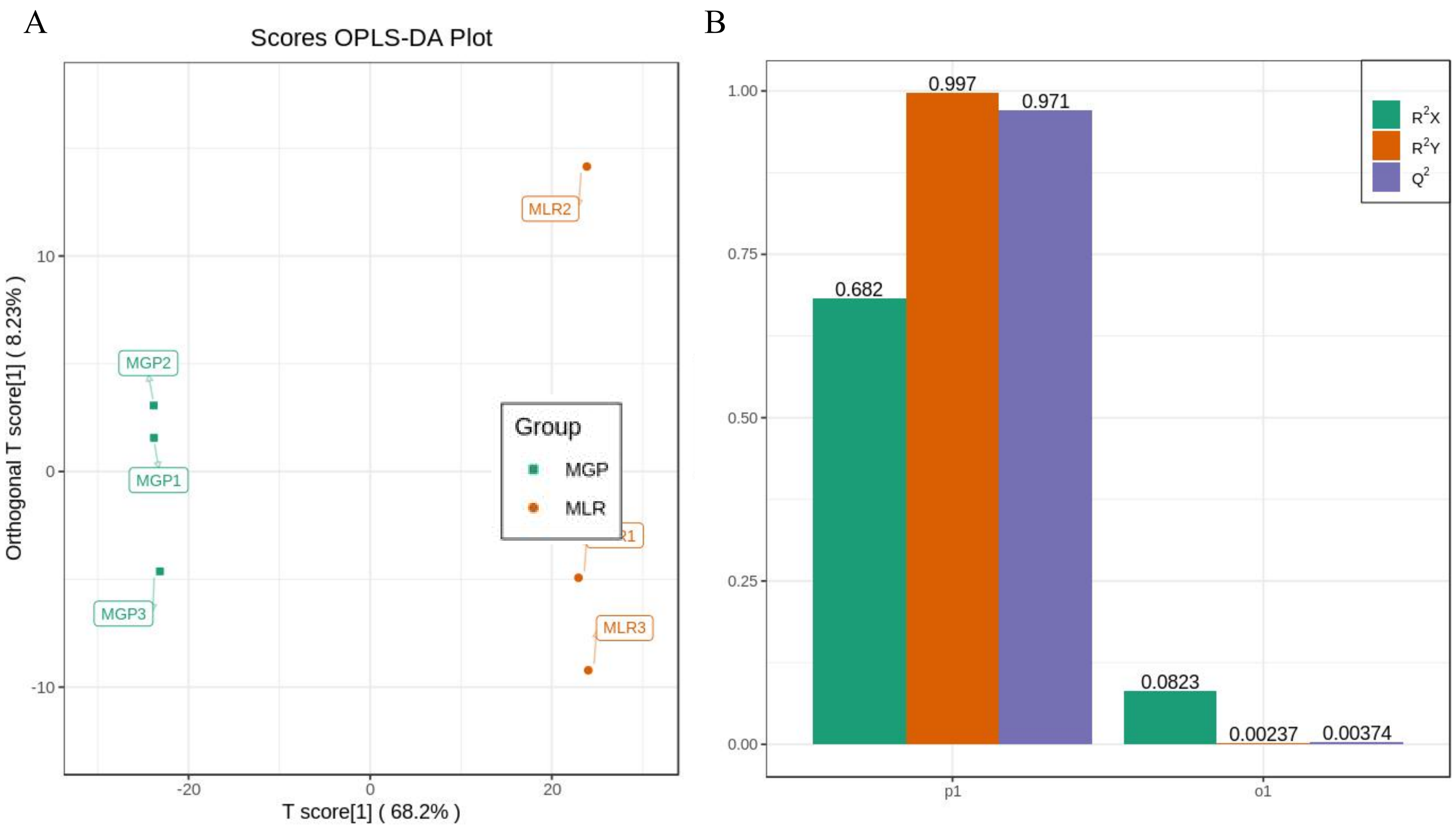

Figure S2-3. Orthogonal partial least squares-discriminant analysis (OPLS-DA) (A) and 200-response sorting tests of MGP_vs_MLR (B).

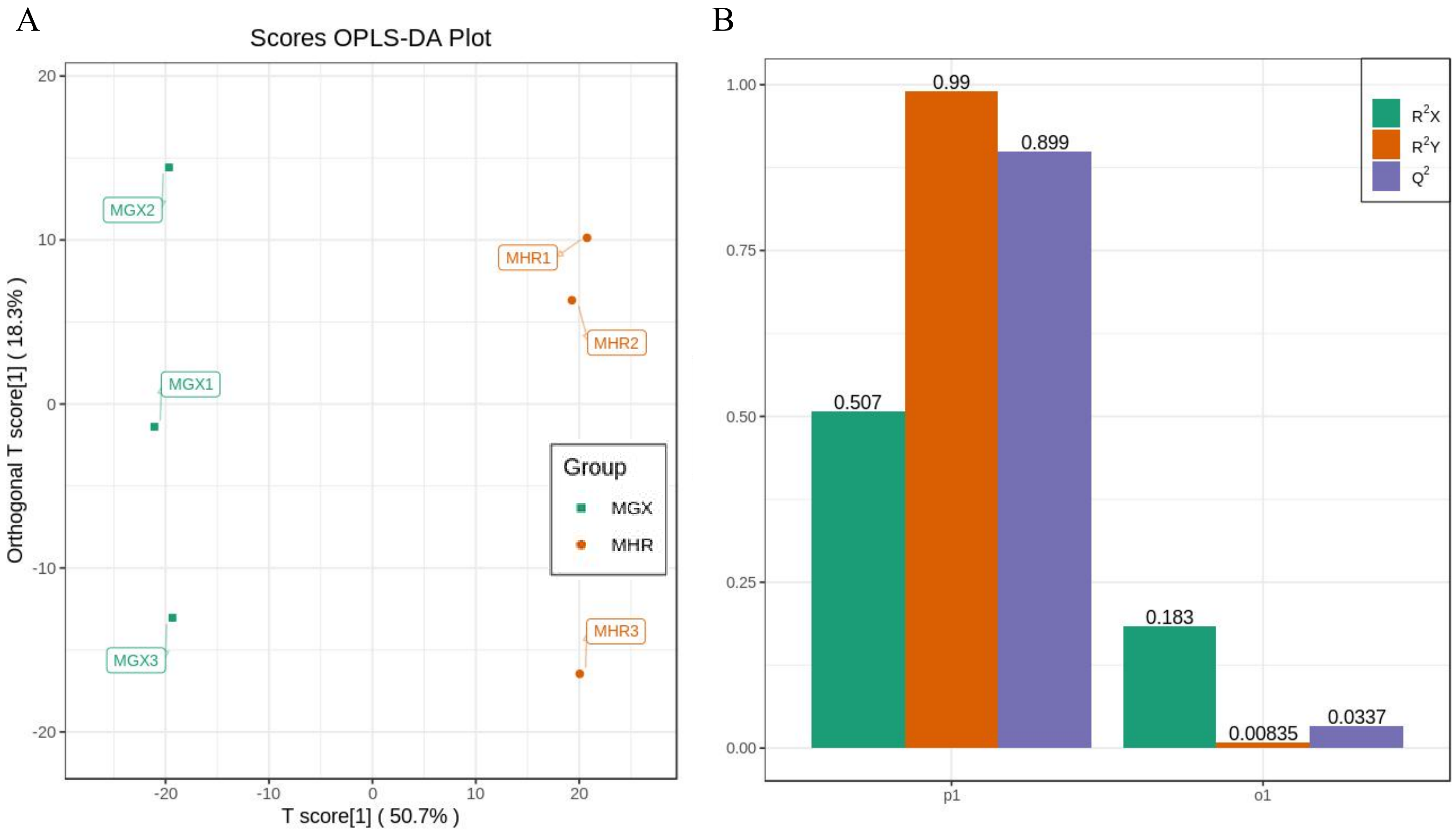

Figure S2-4. Orthogonal partial least squares-discriminant analysis (OPLS-DA) (A) and 200-response sorting tests of MGX_vs_MHR (B).

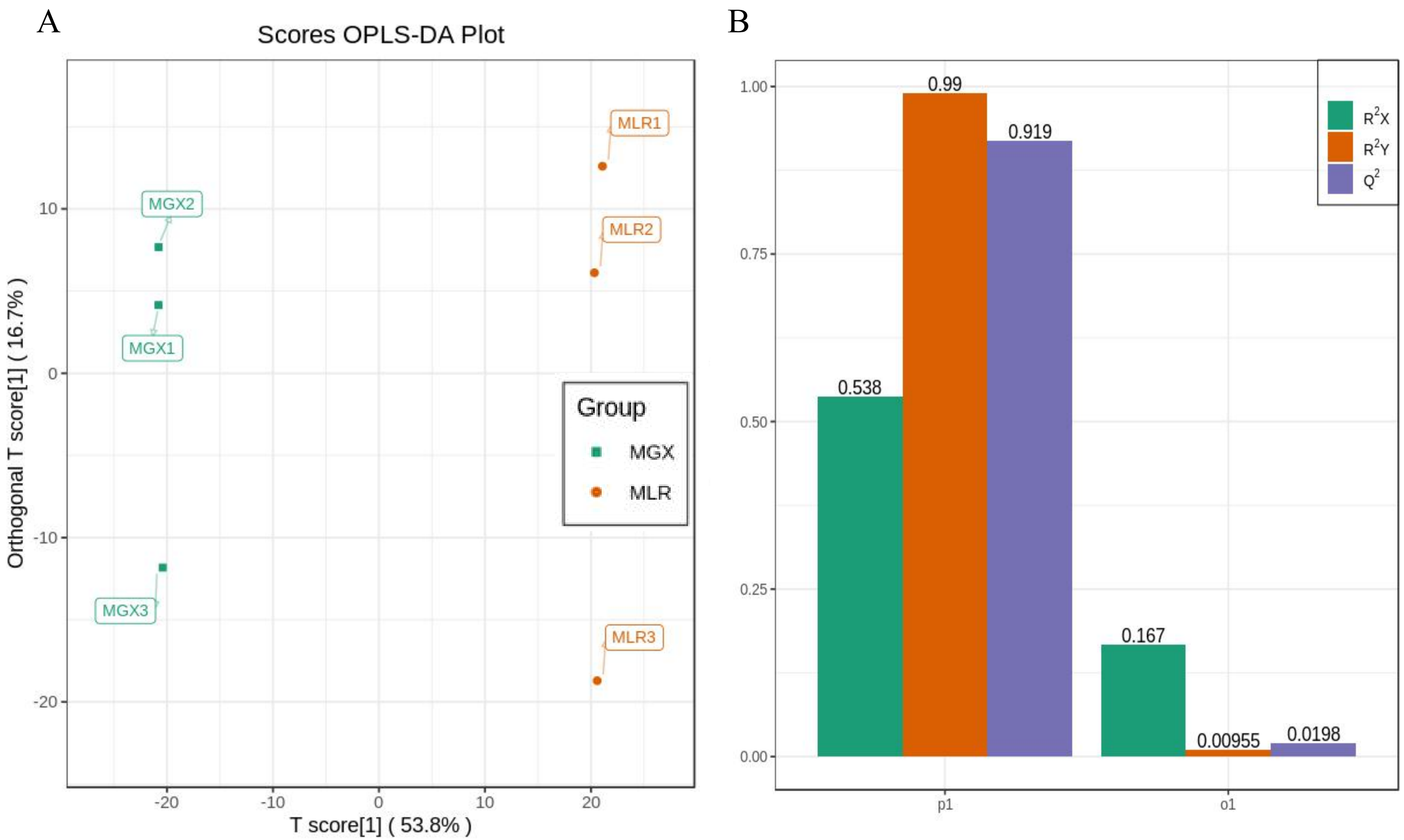

Figure S2-5. Orthogonal partial least squares-discriminant analysis (OPLS-DA) (A) and 200-response sorting tests of MGX_vs_MLR (B).
