# Supplementary material for: Combined Widely Targeted Metabolomic, Transcriptomic, and Spatial Metabolomic Analysis Reveals the Potential Mechanism of Coloration and Fruit Quality Formation in Actinidia chinensis cv. Hongyang
Source: Foods. 2024 Jan 11;13(2):233. doi: 10.3390/foods13020233 (PMC10814455; doi:10.3390/foods13020233)
Supplement: Supplementary file 1 [file foods-13-00233-s001.zip › Figure S3.pdf]

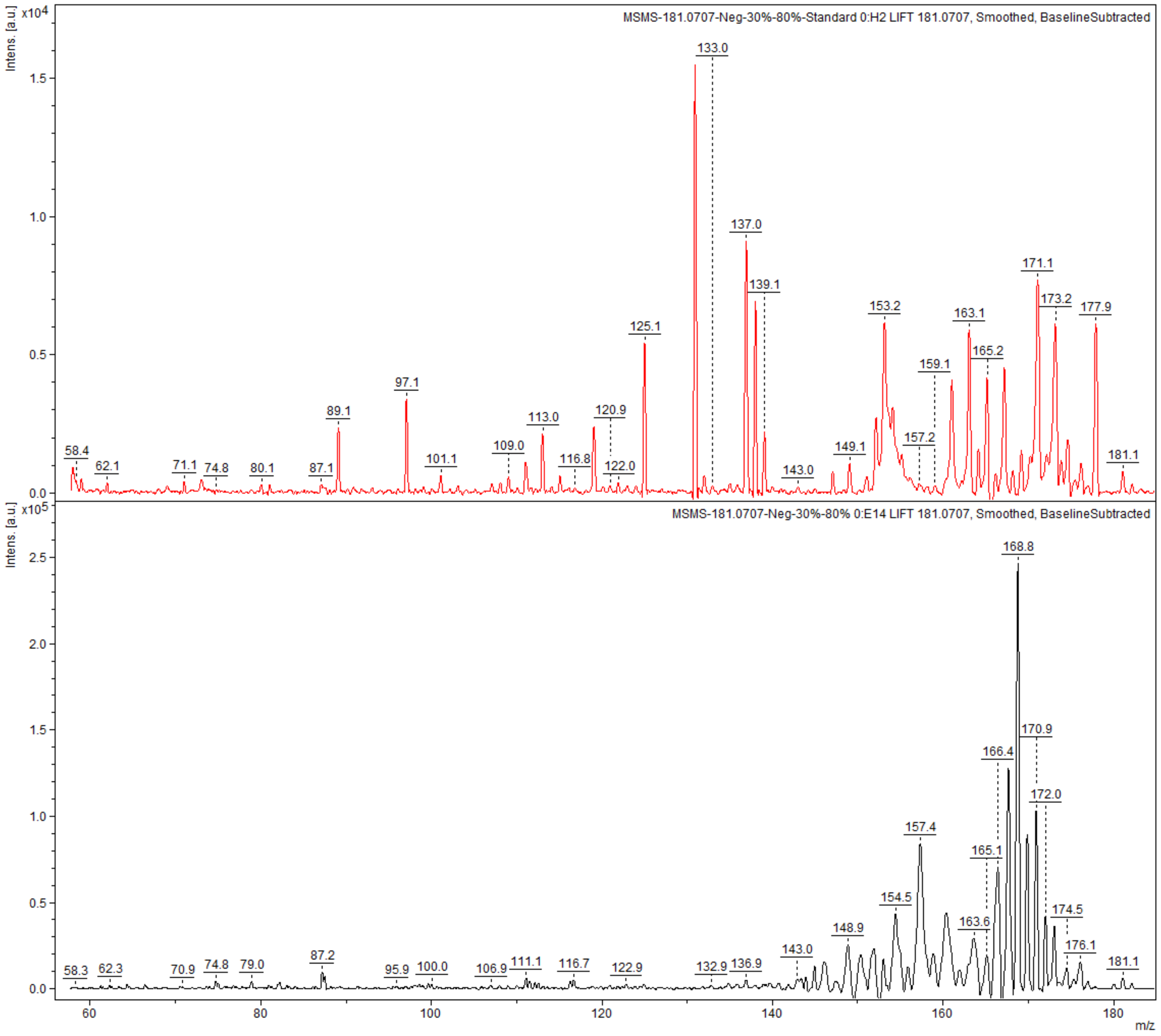

Figure S3-1. Secondary spectrum of sorbitol

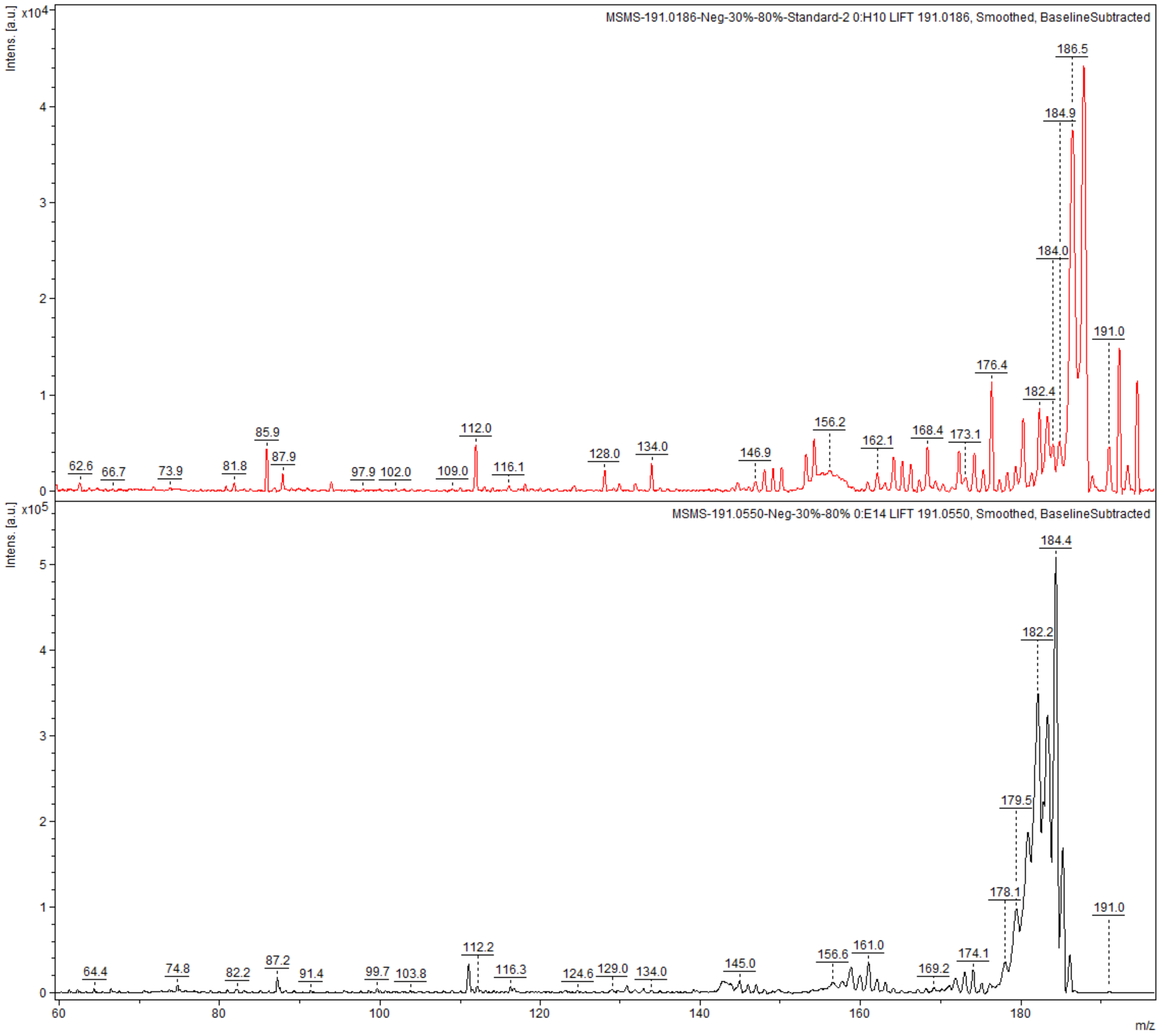

Figure S3-2. Secondary spectrum of citric acid

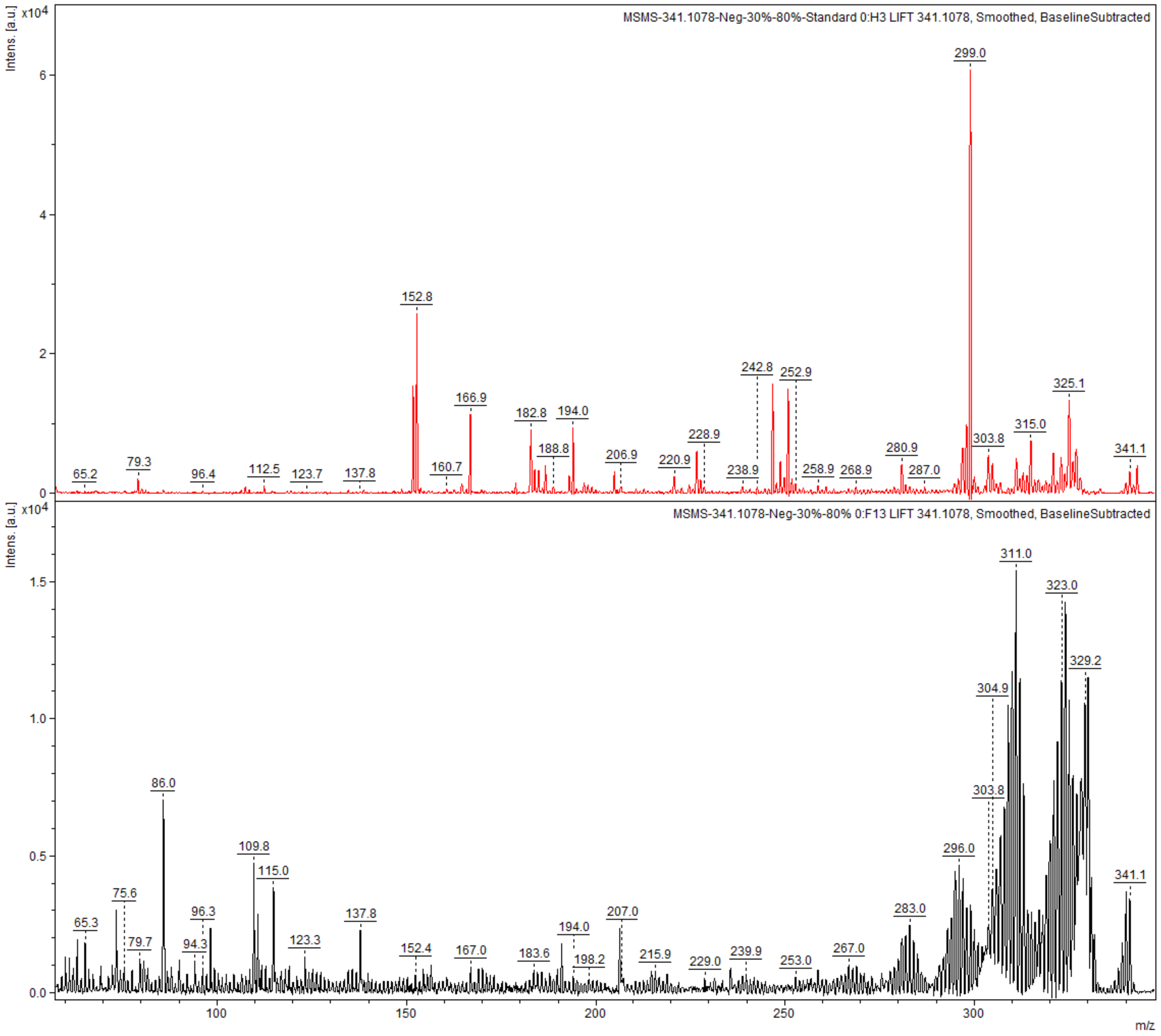

Figure S3-3. Secondary spectrum of sucrose

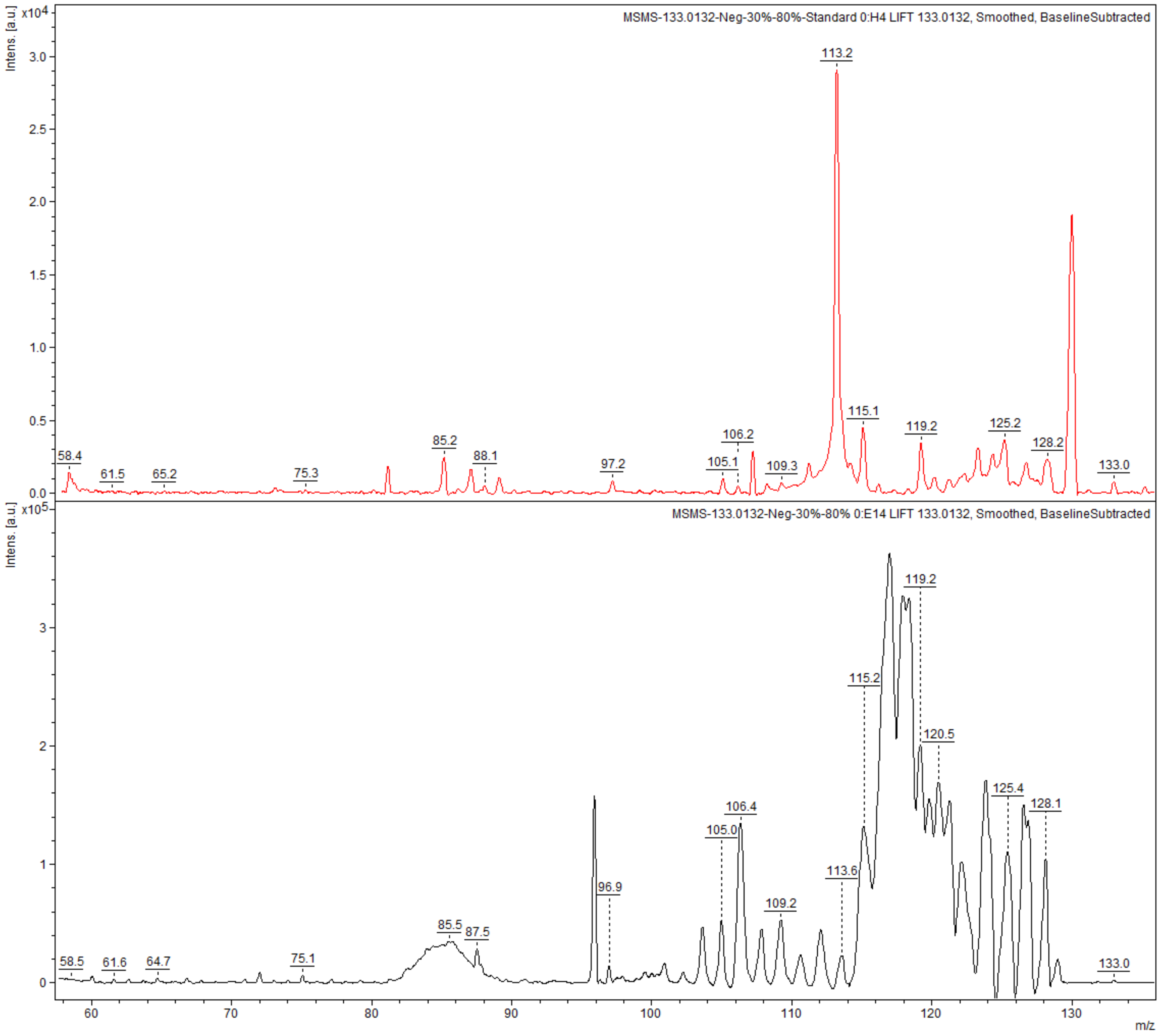

Figure S3-4. Secondary spectrum of malic acid

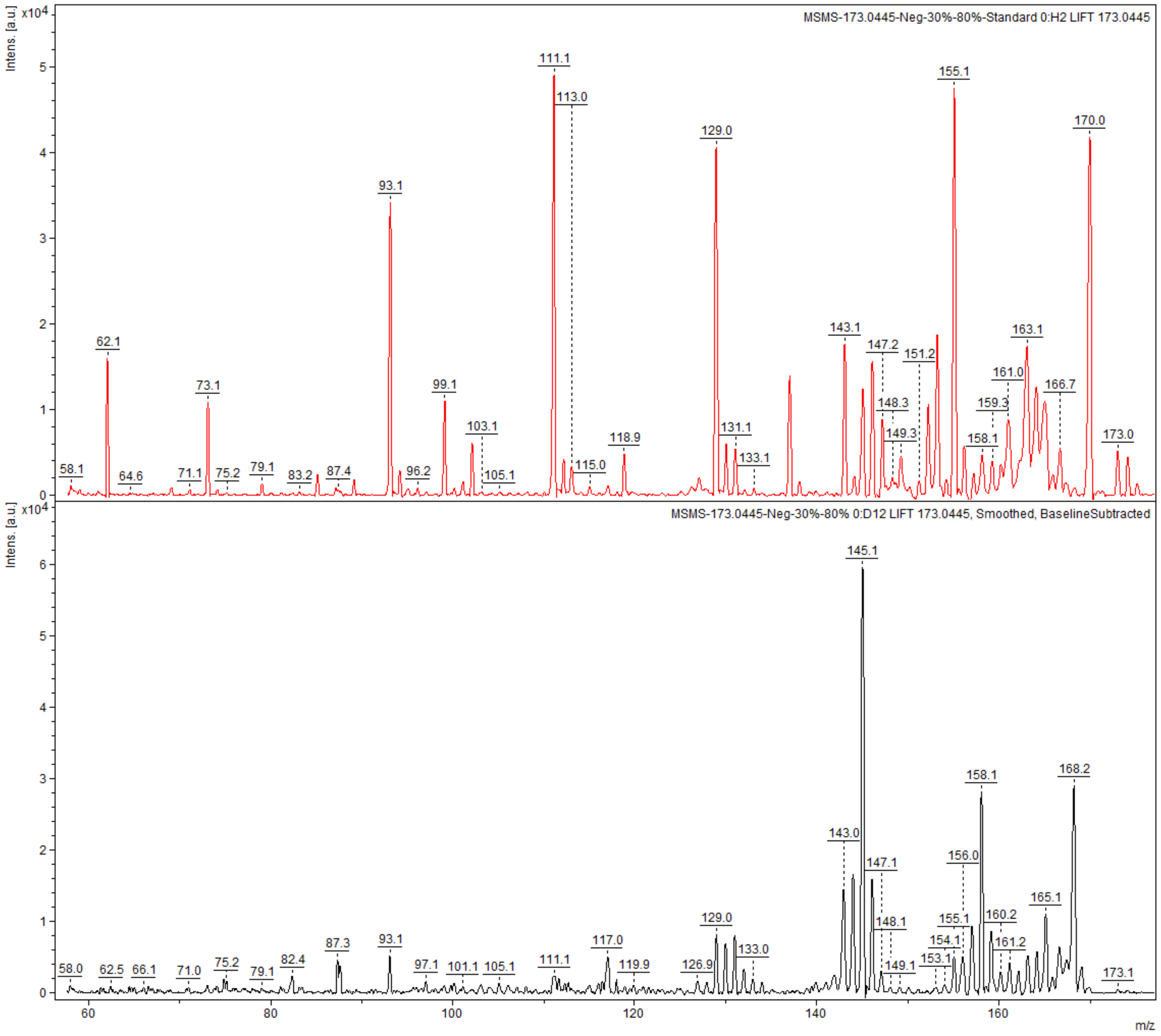

Figure S3-5. Secondary spectrum of shikimic acid

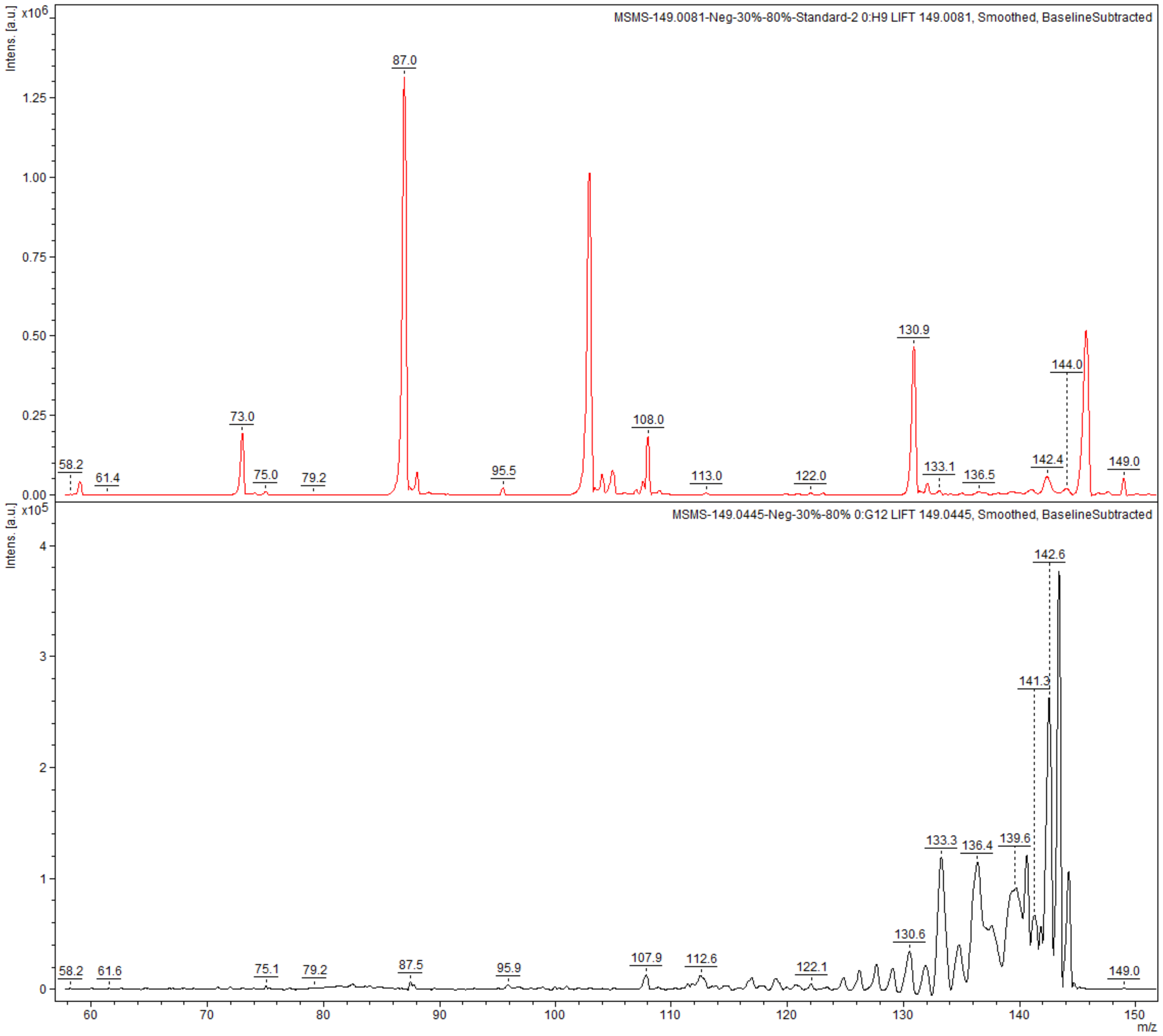

Figure S3-6. Secondary spectrum of tartaric acid

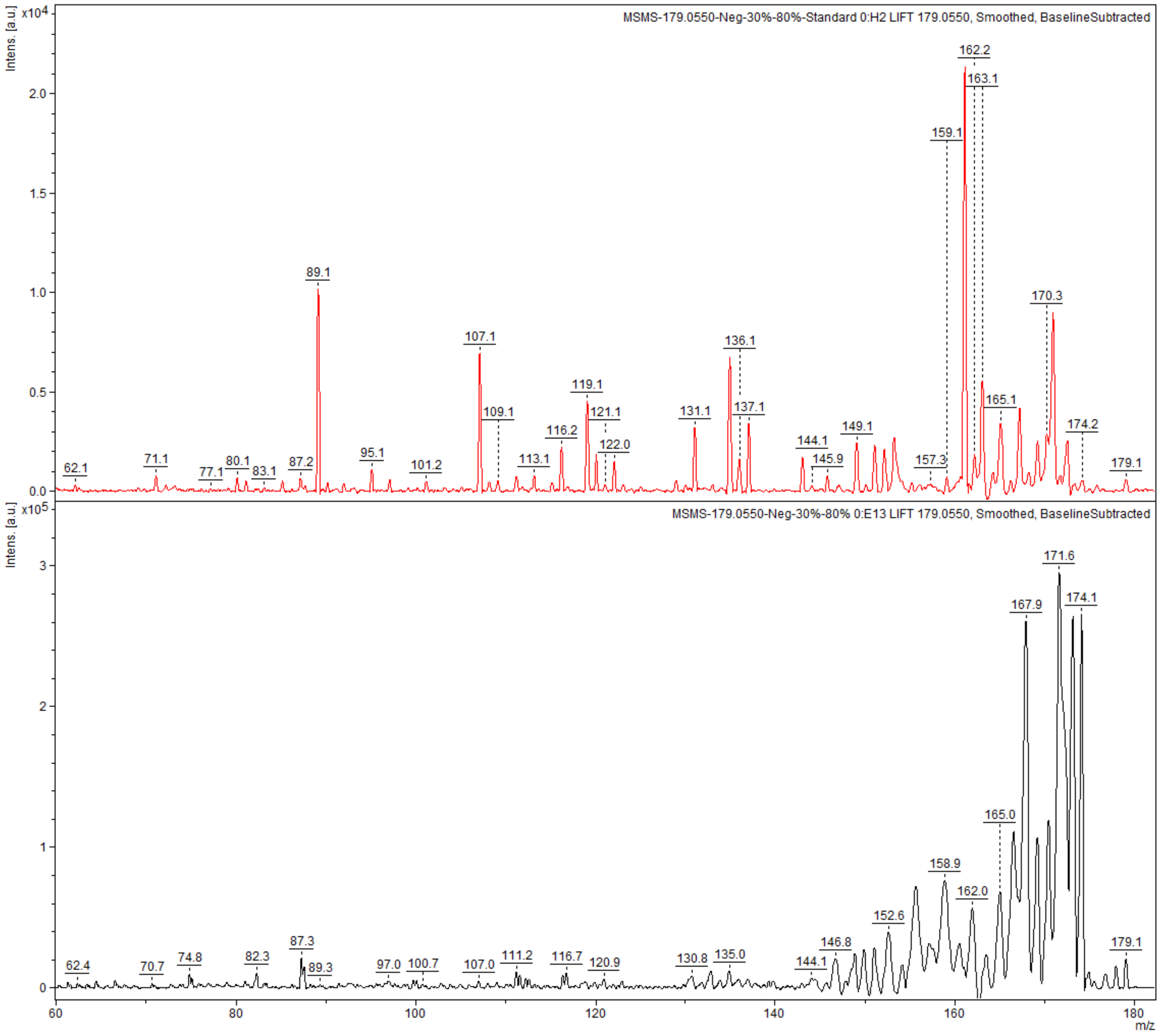

Figure S3-7. Secondary spectrum of glucose
