# Supplementary material for: Combined Widely Targeted Metabolomic, Transcriptomic, and Spatial Metabolomic Analysis Reveals the Potential Mechanism of Coloration and Fruit Quality Formation in Actinidia chinensis cv. Hongyang
Source: Foods. 2024 Jan 11;13(2):233. doi: 10.3390/foods13020233 (PMC10814455; doi:10.3390/foods13020233)
Supplement: Supplementary file 1 [file foods-13-00233-s001.zip › Table S1.pdf]

Table S1. The Primer Information of 12 Candidate Genes and Internal Control Genes Used for qRT-PCR Analysis

Gene	Forward and Reverse primer sequences	Annealing (℃)	ProdSize
bZIP44	F:AGGAAGAGAAAGAGGAAGAGGATGG	57.2	141
	R:AATGCCCGTAATGATCTGACTGTTC	56.8	
DELLA	F:TGACTGGCACCAAGGCTGAC	55.4	116
	R:AACACCGCACTTCTGGGAGAG	54.0	
WD-40	F:GGAGACGGCGATGAAGAACAAC	54.5	112
	R:AACTCGGAACACCTTGACCAAATC	55.5	
MYB5b	F:GTCCGCACCAAGGCATCAC	54.0	133
	R:TCACCAGGTCAGCCCAATCG	55.5	
BHLH052	F:CGGCAATGATCCTATGGTTCTGG	59.5	95
	R:CCTTGGCAGACTCGTCCTCAC	60.5	
MADS	F:AGAACAAGATAAACAGGCAGGTGAC	58.2	150
	R:TGGAGGTGGAGCAGAACTCATAG	59.2	
4CL	F:TGCCGCTCTTCCACATCTACTC	60.4	132
	R:TCGTGACCTTATACCGCTCCATC	59.8	
GATA9	F:TCTCCGTTCCGTGCGAAGAC	59.7	127
	R:TTGTGCGATGACTCCTTGTTGAC	61.5	
CYP78A9	F:AGGAACAGATACCGTGGCAGTC	58.5	85
	R:ATCGTGGACCCTTGATTGAACATC	59.5	
WRKY52	F:TCGTTGTTGCTATGCCCTTTGG	60.2	113
	R:ATGGGAGAATTGGTGACTGAAGTTG	60.5	
ERF109	F:CGAGAATGGATGATGACCTTGATGG	60.5	130
	R:GAGAGCGAGAGAAAGAGTGAGAAAC	58.5	
CYP82D47	F:AACGAGCGGTGGTGGTGAG	59.5	118
	R:GCATAGTTGTAGCCGAGGTGTTG	59.2	
AcActin	F:ATGGCATGAGGGAGGGCATACC	61.5	95
	R:GCTAGTGGTCGTACAACTGGGATTG	60.3	
