# Supplementary material for: Combined Widely Targeted Metabolomic, Transcriptomic, and Spatial Metabolomic Analysis Reveals the Potential Mechanism of Coloration and Fruit Quality Formation in Actinidia chinensis cv. Hongyang
Source: Foods. 2024 Jan 11;13(2):233. doi: 10.3390/foods13020233 (PMC10814455; doi:10.3390/foods13020233)
Supplement: Supplementary file 1 [file foods-13-00233-s001.zip › Table S2.pdf]

Table S2. The Basis Statistics of RNA-seq Data of 12 cDNA Libraries

Sample	Raw Reads	Clean Reads	Clean Base(G)	Error Rate(%)	Q20(%)	Q30(%)	GC Content(%)
TGP1	47 114 362	42 376 466	6.36	0.03	97.72	93.28	46.18
TGP2	57 815 004	56 205 668	8.43	0.03	97.94	93.85	45.51
TGP3	52 418 428	46 497 932	6.97	0.03	97.69	93.22	46.24
TGX1	52 555 198	51 317 602	7.7	0.03	98.03	94.12	45.42
TGX2	65 626 770	64 261 186	9.64	0.03	98.05	94.1	45.75
TGX3	46 426 264	43 081 006	6.46	0.03	97.4	93.03	46.26
THR1	47 676 968	44 571 254	6.69	0.03	97.55	93.4	46.71
THR2	47 628 554	43 325 780	6.5	0.03	97.58	93.4	46.83
THR3	51 467 092	50 249 856	7.54	0.03	98.02	94.04	45.66
TLR1	43 498 248	40 563 518	6.08	0.03	97.61	93.51	46.65
TLR2	54 060 500	50 650 182	7.6	0.03	97.4	93.06	46.69
TLR3	47 587 830	44 573 178	6.69	0.03	97.54	93.28	46.84
